# Society Meetings

**Published:** 1903-01

**Authors:** 


### SOCIETY MEETINGS.

The Erie County Medical Association held its quarterly meeting in Buffalo at the University Club, Monday evening, December 8, 1902, under the presidency of Dr. J. W. Grosvenor, of this city, The scientific program was as follows: Presentation of cases and specimens; Paper: Hypertrophy of the Prostate, Marcel Hartwig. Leaders of discussion, W. C. Phelps, E. J. Meyer. Discussion of the question: Is a cycloplegic necessary for refractive work? Affirmative, A. G. Bennett; negative, E. E. Blaauw. General discussion. Dr. Jacob S. Otto is the secretary.

---

The Fourteenth International Congress of Medicine will be held in Madrid, Spain, from April 23 to 30, 1903, under the patronage of their Majesties, the King of Spain and the Queen-Mother.

The President of the Congress is Professor Julian Calleja y Sanchez, the General Secretary is Dr. Angel Fernandez-Caro, and the General Treasurer is Prof. Jose Gomez Ocana.

The preliminary statements of regulations and program have been issued, and announce that members of the congress will be physicians, pharmacists, dentists, veterinary surgeons, and other persons working at branches of medical science, both Spaniards and foreigners, who have entered their names and paid their subscriptions. Other persons, who possess scientific or professional titles, and who wish to take part in the work of the congress may share in it under the above conditions.

The subscription is thirty pesetas, and this sum must be paid before the opening of the congress to the general secretary, Faculty of Medicine, Madrid. A card of membership will be sent to the subscriber.

Until March 20, 1903, subscriptions may be paid to the secretary of the national committee of the subscriber, but after that date subscriptions must be paid directly to the general secretary at Madrid.

Blank forms of application addressed to the Bureau des Logements in Madrid, forms of application for those who desire to read papers at the Congress, as well as other literature bearing on the subject may be obtained upon request from the undersigned.

Members will receive a summary of the proceedings of the congress and a full report of the work of the particular section which they join.

The official languages of the congress will be Spanish, French, English, German and Italian.

Papers must be sent to the general secretary on or before January 1, 1903, to be certain of a place in the order of business.

Papers presented later will be considered after the discussion of those regularly announced.

Communications should be accompanied by a short abstract, which will be printed and distributed among the members of the congress.

J. H. Huddleston, Secretary American Committee, 126 West 85th St., New York City.

---

THE American Medical Association will hold its fifty-fourth annual meeting at New Orleans, May 5, 6, 7 and 8, 1903. The officers are as follows: president, Frank Billings, Illinois; first vice-president, J. A. Witherspoon, Tennessee; second vice-president G. F. Comstock, New York; third vice-president, C. R. Holmes, Ohio; fourth vice-president, James H. Dunn, Minnesota; secretary-editor, George H. Simmons, Illinois; treasurer, Henry P. Newman, Illinois; chairman committee of arrangements, Isadore Dyer, 124 Baronne Street, New Orleans; board of trustees, Miles F. Porter, Ft. Wayne, 1903; E. Fletcher Ingals, Chicago, 1903; W. L. Rodman, Philadelphia, 1903; W. W. Grant, Denver, 1904; John F. Fulton, St. Paul, 1904; T. J. Happel, chairman, Trenton, Tenn., 1904; E. E. Montgomery, secretary, Philadelphia, 1905; H. L. E. Johnson, Washington, D.C., 1905; A. L. Wright, Carroll, Iowa, 1905; judicial council, term expires 1903: F. H. Wiggin, secretary, New York; G. B. Gillespie, Tennessee; D. C. Peyton, Indiana. Term expires 1904: T. C. Martin, Ohio; J. B. Roberts, Pennsylvania; Christopher Tompkins, Virginia. Term expires 1905: Philip Marvel, chairman, New Jersey; George Cook, New Hampshire; N. S. Davis, Jr., Illinois; oration on medicine, J. M. Anders, Philadelphia; oration on surgery, A. F. Jonas, Omaha; oration on state medicine, Wm. H. Welch, Baltimore.

*Officers of Sections.*—Practice of medicine—chairman, W. S. Thayer, Baltimore; secretary, James B. McElroy, Stoval, Miss.; executive committee, George Dock, Ann Arbor; J. M. Anders, Philadelphia; Frank A. Jones, Memphis.

Obstetrics and diseases of women—chairman, A. Palmer Dudley, New York; secretary, C. L. Bonifield, Cincinnati; executive committee, W. E. B. Davis, Birmingham; Henry P. Newman, Chicago; J. H. Carstens, Detroit.

Surgery and anatomy—chairman, James E. Moore, Minneapolis; secretary, John C. Munro, Boston; executive committee, H. O. Walker, Detroit; A. J. Ochsner, Chicago; DeForest Willard, Philadelphia.

Hygiene and sanitary science—chairman, H. M. Bracken, Minneapolis; secretary, G. T. Swarts, Providence; executive committee, Arthur R. Reynolds, Chicago.

Ophthalmology — chairman, John E. Weeks, New York; secretary, Frank Todd, Minneapolis; executive committee, H. V. Würdemann, Milwaukee; J. A. Lippincott, Pittsburg; Frank Allport, Chicago.

Diseases of children—chairman, John C. Cook, Chicago; secretary, Thos. S. Southworth, New York City; executive committee, Edwin Rosenthal, Philadelphia; Samuel W. Kelley, Cleveland; H. M. McClanahan, Omaha.

Stomatology—chairman, M. L. Rhein, New York; secretary, Eugene S. Talbot, Chicago; executive committee, M. H. Fletcher, Cincinnati; R. R. Andrews, Cambridge; A. H. Peck, Chicago.

Nervous and mental diseases—chairman, F. W. Langdon, Cincinnati; secretary, F. Savary Pearce, Philadelphia; executive committee, Hugh T. Patrick, Chicago; H. A. Tomlinson, St. Peter, Minn.; Richard Dewey, Wauwatosa, Wis.

Cutaneous medicine and surgery—chairman, John A. Fordyce, New York; secretary, R. R. Campbell, Chicago; executive committee, L. Duncan Bulkley, New York; W. L. Baum, Chicago; Henry W. Stelwagon, Philadelphia.

Laryngology and otology—chairman, Geo. L. Richards, Fall River; secretary, J. F. Barnhill, Indianapolis; executive committee, C. R. Holmes, Cincinnati; J. N. Mackenzie, Baltimore; G. Hudson Makuen, Philadelphia.

Materia medica, pharmacy and therapeutics—chairman, Solomon Solis-Cohen, Philadelphia; secretary, C. S. N. Hallberg, Chicago; executive committee, Leon L. Solomon, Louisville; N. S. Davis, Jr., Chicago; George F. Butler, Alma, Mich.

Pathology and physiology—chairman, Victor C. Vaughan, Ann Arbor; secretary, Joseph McFarland, Philadelphia; executive committee, W. S. Hall, Chicago; L. Hektoen, Chicago; Frank B. Wynn, Indianapolis.

New Orleans is a most interesting and hospitable city and the association is fortunate in its selection as a place of meeting. In May it may be said to be at its best in its garb of spring, with its magnolias in blossom perfuming the air with sweetest odors, and all nature is in her best attire to welcome visitors.

The chairman of the committee of arrangements, Dr. Isadore Dyer, is well known to the profession as a capable executive officer, and has put the machinery in motion for a splendid business and social reception of the association.

---

The American Röntgen Ray Society held its third annual meeting at Chicago, December 10 and 11, 1902, at which Dr. John C. Pitkin, of Buffalo, read a paper entitled, Equal potential surfaces in the *x*-ray field.

---

The Medical Society of the County of Erie will hold its 82d annual meeting in the parlors of the Y. M. C. A. Building, corner Mohawk and Pearl Streets, beginning at 10 o'clock a.m.,

Tuesday, January 13, 1903, under the presidency of Dr. Walden M. Ward, of North Collins. The secretary, Dr. F. C. Gram, is preparing the program, which will include some interesting features.

---

THE Buffalo Academy of Medicine held meetings during the month of December, 1902, as follows:

*Section on Surgery.*— Tuesday evening, December 2. Program: To what extent may the female genitals be saved from mutilation? J. Henry Dowd; The use of silver leaf in the treatment of skin ulcers, David E. Wheeler; Are antiseptics of any value in hand disinfection? E. R. McGuire. Discussion: Drs. Park, Parmenter, E. A. Smith and others.

*Section on Medicine.*—Tuesday evening, December 9. Program: Pulsations of the thoracic wall, with report of a case of pulsating hemothorax. Report of a case of eventration of the diaphragm, Joseph Sailer, Philadelphia; Nephritis in infants, Charles Sumner Jones.

*Section on Pathology.*—Tuesday evening, December 16. Program: The prevalence and geographic distribution of hookworm disease (uncinariasis) in the United States, Charles Wardell Stiles, zoologist to the United States Public Health and Marine Hospital Service, Washington, D.C.

*Section on Obstetrics and Gynecology.*—Tuesday evening, December 23. Program: Indications for inducing abortion, Marcel Hartwig.

---

THE Medical Society of the State of New York will hold its 97th annual meeting at Albany, Tuesday, Wednesday and Thursday, January 27, 28 and 29, 1903, under the presidency of Dr. Henry Reed Hopkins, of Buffalo. The committee of arrangements, under the chairmanship of Dr. Ernest Wende, is preparing an elaborate program which will soon be published.

